# Using Twitter for research in psychiatry

**DOI:** 10.1192/j.eurpsy.2023.188

**Published:** 2023-07-19

**Authors:** M. Pinto da Costa

**Affiliations:** ^1^Institute of Psychiatry, Psychology & Neuroscience, King’s College London, London, United Kingdom; ^2^Institute of Biomedical Sciences Abel Salazar, University of Porto, Porto, Portugal

## Abstract

**Abstract:**

Social media offers a unique opportunity to examine behaviors based on real-time objective data. For example, all public tweets that include the selected keywords can be collated. These offer vast opportunities to research attitudes towards mental health and mental illness in the general population, based on the tweets content. The tweet text, the date, the geolocation and international timestamp of when they were published, and the number of retweets and likes generated are informative data that can be extracted. Content analysis can be conducted using manual or machine learning approaches. Different examples of the use of Twitter for research in psychiatry will be presented and discussed in this session.

**Disclosure of Interest:**

None Declared

